# Molecular Recognition Mechanism of Key VOCs by Odorant-Binding Proteins in the Western Corn Rootworm (*Diabrotica virgifera virgifera*)

**DOI:** 10.3390/insects17060595

**Published:** 2026-06-05

**Authors:** Huijie Zhao, Zongpei Zhao, Yaru Zhao, Sizhu Zheng, Lei Wang, Yujie Lu

**Affiliations:** 1 School of Grain Science and Technology, Jiangsu University of Science and Technology, Zhenjiang 212100, China; zhaohuijieuuu@163.com (H.Z.); zongpeizhao@just.edu.cn (Z.Z.); zhaoyarunitu@163.com (Y.Z.); 2 Animal, Plant and Food Inspection Center, Nanjing Customs, Nanjing 210001, China; zhengsizhu@126.com

**Keywords:** *Diabrotica virgifera virgifera*, odorant-binding proteins, volatile organic compounds, molecular docking

## Abstract

The western corn rootworm (*Diabrotica virgifera virgifera*) relies on odorant-binding proteins (OBPs) to locate maize hosts and mates via volatile organic compounds (VOCs). However, the molecular recognition mechanisms of specific attractants, such as (E)-β-caryophyllene, 6-methoxy−2-benzoxazolinone (6-MBOA), and the sex pheromone 8R-methyl−2R-decyl propanoate (2R,8R-MDP), remain elusive. Here, we integrated phylogenetic analysis, AlphaFold2 structural prediction, molecular docking, molecular dynamics (MD) simulations, and in vitro fluorescence competitive binding assays to characterize the binding specificity of DvirOBPs toward these key ligands. Pan-family screening identified DvirOBP54b as possessing the highest ligand-binding specificity, resolving its evolutionary divergence from its tandem duplicative paralog DvirOBP54a. Structural and dynamic analyses revealed that DvirOBP54b binding to (E)-β-caryophyllene and 2R,8R-MDP is predominantly driven by hydrophobic interactions within a core pocket (Phe7, Phe69, Ile70, Ala121). Conversely, its interaction with 6-MBOA is further stabilized by a specific hydrogen bond at Thr66. Dynamic trajectories confirmed the high stability of the DvirOBP54b complexes, while in vitro assays validated the strong binding affinities toward the core host-derived volatiles. These findings elucidate the structural basis of VOC-mediated olfactory recognition in *D. v. virgifera*, providing critical molecular targets for developing high-efficiency attractants and novel pest management strategies.

## Simple Summary

The western corn rootworm is a severe pest that destroys corn crops worldwide, causing enormous economic losses. To find food and mates, these insects rely on a highly sensitive sense of smell, using special proteins to capture scent molecules from the environment. This study aimed to discover exactly how these scent-capturing proteins recognize specific odors released by corn roots. By combining computer simulations with laboratory experiments, we showed that odorant-binding proteins are highly specialized in binding to key corn odors. We predicted the three-dimensional structural models of these proteins, demonstrating how their internal pocket acts like a lock to perfectly fit odor molecule keys, primarily via hydrophobic interactions. Understanding this microscopic smelling mechanism provides the essential blueprint for developing new, environmentally friendly pest management strategies. By creating advanced traps or odor-blocking chemicals based on these structural findings, we can disrupt the insect’s ability to locate crops, ultimately protecting global food supplies and reducing the reliance on traditional chemical pesticides.

## 1. Introduction

The western corn rootworm (*Diabrotica virgifera virgifera*, WCR) is recognized as a globally important quarantine pest causing severe damage to maize [1]. *D. v. virgifera* larvae damage maize roots, which become susceptible to pathogen infection, causing root rot and increasing the risk of lodging, which hinders mechanical harvesting and further exacerbates yield losses. Adults feed on maize pollen, silks, kernels, and leaves, disrupting pollination and kernel development, thereby reducing both crop quality and yield [2]. In addition, western corn rootworms adults act as vectors of maize chlorotic mottle virus (MCMV). In the United States, annual yield losses caused by *D. v. virgifera* amount to as much as $2 billion, whereas potential economic losses in Europe exceed €400 million [3]. Therefore, strengthening the quarantine, inspection, detection, and monitoring of the western corn rootworm is essential for ensuring the healthy development of the maize industry.

Herbivorous insect pests locate host plants by recognizing volatile organic compounds (VOCs) released by plants. Their host-finding behavior shifts from directed movement guided by general environmental cues to the precise identification of host-specific volatile signals [4]. This plant–insect chemical communication mechanism provides a robust theoretical basis for insect behavioral manipulation. Carbon dioxide (CO_2_), which is continuously released by plant roots and diffuses rapidly in the soil matrix, acts as a universal attractant for soil-dwelling insects [5]. However, CO_2_ is ubiquitous in soil environments and lacks host-specificity; therefore, it can hardly act as a reliable signal for precise host location when used alone. Instead, the host-seeking behavior of soil-dwelling insects mostly depends on supplementary chemical cues [6]. Research has demonstrated that WCR exhibits highly specific chemotaxis toward maize-emitted VOCs, and multiple key semiochemicals that regulate its host-finding behavior have been functionally identified. Compared to CO_2_ alone, first-instar WCR larvae show a six times stronger behavioral response to the mixture of CO_2_ and host volatiles [7]. Among these active compounds, 6-methoxy−2-benzoxazolinone (6-MBOA) has been confirmed as a key active component [7,8]. In addition, (E)-β-caryophyllene is released by maize roots in response to herbivore attack; this compound is not only highly attractive to rootworm larvae but also exhibits favorable diffusion properties in the soil matrix [9]. These findings confirm that the host location of WCR is mainly mediated by the olfactory recognition of plant volatiles. In addition, the sex pheromone, 8R-methyl−2R-decyl propanoate (2R,8R-MDP), produced by female WCR adults, elicits a strong attractive response in conspecific males [10]. The integrated application of these semiochemicals provides a promising strategy for the efficient monitoring and sustainable management of this destructive agricultural pest.

Insects rely on a highly sensitive olfactory system to detect and discriminate volatile compounds. Central to this process are odorant-binding proteins (OBPs), which are abundantly located in the sensillar lymph of the antennae. OBPs specifically bind hydrophobic odor molecules and transport them to olfactory receptor neurons, thus initiating signal transduction and triggering downstream olfactory behaviors. The selective binding of different OBPs to odorants forms the molecular basis for insect discrimination of complex odor environments [11]. Investigating the interactions between OBPs and volatile compounds is crucial not only for elucidating the molecular mechanisms governing ligand binding and release, but also for providing a theoretical foundation for disrupting host-location behavior in pest control strategies. To efficiently identify candidate OBPs involved in these processes, molecular docking has emerged as a powerful and high-throughput approach for the initial screening of extensive odorant libraries. This technique evaluates binding affinity by constructing three-dimensional structures of protein–ligand complexes and identifying potential binding sites [12], significantly accelerating the discovery of functional proteins. However, while molecular docking provides a predictive baseline, these in silico findings must be corroborated by empirical evidence to ensure biological accuracy. Consequently, fluorescence competitive binding assays serve as a critical tool for providing direct experimental evidence of OBP–ligand interactions [13]. By integrating the high-throughput predictive power of molecular docking with the precise validation of in vitro fluorescence assays, a more comprehensive and reliable understanding of the molecular recognition mechanisms underlying insect olfaction can be achieved.

In this study, high-confidence three-dimensional structures of target OBPs were constructed by using AlphaFold2-based modeling. (E)-β-caryophyllene, 6-MBOA and 2R,8R-MDP were selected as target ligands. The binding affinities between DvirOBPs and the target ligands were analyzed by molecular docking. To validate these in silico predictions, in vitro fluorescence competitive binding assays were conducted using N-phenyl−1-naphthylamine (1-NPN) as a fluorescent probe for the two key host-derived volatiles (6-MBOA and (E)-β-caryophyllene). The experimental results confirmed that DvirOBP54b binds strongly to both ligands, with dissociation constants (K_i_) consistent with the predicted binding free energies, thereby supporting the reliability of our computational models. Furthermore, the specific interaction patterns between DvirOBP54b and the three ligands were systematically analyzed. These findings provide integrated structural and experimental insights into the olfactory molecular recognition mechanisms in WCR.

## 2. Materials and Methods

### 2.1. Phylogenetic Analysis of the OBP Gene Family

Based on previously published studies [14,15], a total of 140 OBP sequences from *D. v. virgifera*, 47 from *Tribolium castaneum*, and 19 from *Tenebrio molitor* were retrieved in this study. Subsequently, all sequences were aligned using MAFFT (v7.490, Osaka University, Osaka, Japan) with the “–auto” strategy for multiple sequence alignment. Based on the alignment results, a maximum likelihood phylogenetic tree was constructed using MEGA11 (Pennsylvania State University, University Park, PA, USA). Node support was assessed using the ultrafast bootstrap (UFBoot) method with 1000 replicates, with other parameters set to default values. The inferred phylogenetic tree was then visualized and annotated using the online platform iTOL v6 (EMBL, Heidelberg, Germany; https://itol.embl.de (accessed on 15 December 2025)).

### 2.2. Screening of Characteristic VOC Molecules

Based on previously reported chemical ecology studies of the western corn rootworm, three alternative chemical ligands were selected. Among them, two compounds are larval attractants: 6-MBOA [8] and (E)-β-caryophyllene [9]. The third compound, 2R,8R-MDP, is an adult sex pheromone [10]. The three-dimensional structures of these compounds (in SDF format) were obtained from the PubChem database (https://pubchem.ncbi.nlm.nih.gov (accessed on 30 December 2025)) [16]. Subsequently, the SDF files were converted to PDB format using the open-source cheminformatics tool Open Babel (version 3.1.1, http://openbabel.org (accessed on 30 December 2025)) [17] for subsequent molecular docking analysis in AutoDock Vina (version 1.1.2, The Scripps Research Institute, La Jolla, CA, USA).

### 2.3. Homology Modeling and Molecular Docking

To obtain high-accuracy protein structural models, the three-dimensional structures of DvirOBPs were predicted using ColabFold v1.3.2 (https://github.com/sokrypton/ColabFold (accessed on 13 January 2026)), which is based on the AlphaFold2 algorithm [18,19]. The predictions were run on an NVIDIA A100 GPU, and the resulting structures were subsequently analyzed using PyMOL (v2.6, Schrödinger, LLC, Portland, OR, USA). The binding pockets were identified using the DoGSiteScorer server (https://proteins.plus (accessed on 13 January 2026)). Receptor and ligand preparation were carried out using AutoDock Tools (ADT, v1.5.7, Scripps Research Institute, La Jolla, CA, USA). Then, the polar hydrogen atoms were added, all water molecules were removed, and Kollman charges were assigned, after which the structure was saved in PDBQT format. The three candidate ligands ((E)-β-caryophyllene, 6-MBOA, and 2R,8R-MDP) were processed as follows: water molecules were removed, hydrogen atoms were added, Gasteiger charges were assigned, and all non-ring single bonds were defined as rotatable bonds. Each ligand was then saved in PDBQT format. Molecular docking simulations were performed using AutoDock Vina v1.2.4 (Scripps Research Institute) with the exhaustiveness parameter set to 32. Each ligand was docked independently, yielding nine binding conformations. Docking results were evaluated based on binding free energy (ΔG), and conformations with ΔG < −5.0 kcal/mol were considered to exhibit significant binding activity.

### 2.4. Molecular Dynamics Simulation

Molecular dynamics (MD) simulations of the selected DvirOBP54b–ligand complexes were performed using NAMD 3.0 (University of Illinois at Urbana-Champaign, Urbana, IL, USA) with the CHARMM36m force field. Initial system configurations were built in VMD 1.9.4 (University of Illinois at Urbana-Champaign) and solvated with the TIP3P water model, ensuring a minimum solvent layer thickness of 10 Å. Subsequently, NaCl was added to a final concentration of 150 mM to mimic physiological ionic strength and neutralize the net charge of the system.

System equilibration was performed following a standard multi-step protocol. First, 10,000 steps of energy minimization were conducted using the steepest descent algorithm to eliminate unfavorable atomic contacts. This was followed by a 100 ps equilibration under the canonical (NVT) ensemble, where the temperature was maintained at 300 K using a Langevin thermostat with a damping coefficient of 1.0 ps^−1^. Subsequently, a 500 ps equilibration under the isothermal–isobaric (NPT) ensemble was performed, utilizing a Langevin piston barostat to maintain the pressure at 1 bar.

Production simulations were then carried out under the NPT ensemble for 50 ns with a time step of 2 fs. A cutoff of 10 Å was applied for short-range van der Waals and electrostatic interactions, while long-range electrostatic interactions were treated using the particle mesh Ewald (PME) method. To ensure statistical robustness, three independent replicate simulations were performed for each complex system using different initial random velocities.

The resulting production trajectories were stripped of solvent molecules and counterions, and converted to AMBER-compatible coordinate format via a VMD plugin. These trajectories were subsequently analyzed using the CPPTRAJ module within AmberTools (v18, AmberTools). The root mean square deviation (RMSD) of protein Cα atoms and the root mean square fluctuation (RMSF) of side chain residues were calculated to evaluate structural stability and flexibility. Finally, to quantitatively evaluate the post-simulation energetics, the binding free energies (ΔG) of the complexes were calculated using the Molecular Mechanics/Generalized Born Surface Area (MM/GBSA) method. For this energetic refinement, representative structural snapshots were extracted at regular intervals strictly from the equilibrium phase of the replicated MD trajectories. The final energetic values are reported as the mean ± standard deviation across the three independent replicates.

### 2.5. MM-GBSA Calculations

Binding free energies were calculated using the Molecular Mechanics/Generalized Born Surface Area (MM/GBSA) method [20]. Consistent and compatible force field parameters were used throughout all components. From the equilibrated trajectories of each replicate simulation, 1000 equally spaced conformations were extracted for energy calculation and averaging.

The binding free energy was calculated according to the following equation:

$$\Delta G_{\mathrm{bind}}=\;{\Delta E}_{\mathrm{vdw}}\;+\;{\Delta E}_{\mathrm{ele}}\;+\;{\Delta G}_{\mathrm{polar}\;}+\;{\Delta G}_{\mathrm{nonpolar}}-T\Delta S$$

where ΔE_vdw_ and ΔE_ele_ represent the van der Waals and electrostatic interaction energies, respectively; ΔG_polar_ denotes the polar solvation free energy calculated using the GBneck2 implicit solvent model; ΔG_nonpolar_ represents the nonpolar solvation free energy; and TΔS corresponds to the conformational entropy contribution.

### 2.6. Family-Specific Analysis of DvirOBP–Ligand Binding

To evaluate the binding specificity of DvirOBPs toward different ligands, a specificity index (SI) was introduced as a quantitative metric. The SI is defined as the ratio of the binding free energy of the DvirOBP–ligand complex to the average binding free energy of homologous OBPs within the same family for that ligand:

$$\mathrm{Specificity}\;\mathrm{index}\;=\;{\Delta G}_{\mathrm{target}}/{\Delta G}_{\mathrm{family}\;\mathrm{average}}$$

where ΔG_target_ represents the binding free energy between DvirOBPs and the ligand, and ΔG_family average_ denotes the average binding free energy of family-member OBPs for the same ligand.

### 2.7. Structural Visualization and Interaction Analysis

To elucidate the refined binding modes, the most stable conformations of the DvirOBP54–ligand complexes identified from MD trajectories were visualized. The 3D binding poses and the surrounding amino acid residues were rendered using PyMOL Molecular Graphics System (v2.6, Schrödinger, LLC). Detailed 2D interaction maps, illustrating hydrogen bonding, hydrophobic contacts, and π-stacking, were generated using Discovery Studio (v25.1, BIOVIA, Dassault Systèmes, San Diego, CA, USA).

### 2.8. Protein Production and Purification

The gene sequences of DvirOBPs were amplified from cDNA by PCR, with gene-specific primers designed to incorporate BamHI and XhoI restriction sites. The amplified products were cloned into the pGEX−6P−1 vector, resulting in fusion with a 6× His tag. As the vector itself carries an N-terminal GST tag, the expressed DvirOBPs were fused with both GST and His tags. The recombinant constructs were transformed into *Escherichia coli* BL21 (DE3) cells and selected on LB medium containing ampicillin. A single positive colony was inoculated into 10 mL LB medium supplemented with 100 µg/mL ampicillin and cultured overnight at 37 °C. Subsequently, 3 mL of the overnight culture was transferred into 300 mL LB medium and induced with 0.1 mM isopropyl β-D−1-thiogalactopyranoside (IPTG) (Sigma-Aldrich, St. Louis, MO, USA), followed by incubation at 16 °C for approximately 14 h. The recombinant DvirOBP fusion proteins were purified using affinity chromatography according to the manufacturer’s protocols. Briefly, the clarified supernatant was loaded onto the equilibrated column, followed by extensive washing to remove impurities and subsequent elution with a specific competitive elution buffer. Protein purity was assessed by SDS-PAGE, and protein concentration was determined using the Bradford assay.

### 2.9. Fluorescence Competitive Binding Assays

Following the method described by Wang et al. [21], fluorescence measurements were performed using a Synergy H1 hybrid multi-mode microplate reader (BioTek Instruments, Inc., Winooski, VT, USA) with excitation and emission wavelengths set to 337 nm and 405 nm, respectively. The recombinant proteins (DvirOBP53, DvirOBP54b, and DvirOBP55) were individually diluted to a final concentration of 2 μM in 50 mM Tris-HCl buffer (pH 7.4) (Macklin Biochemical Co., Ltd., Shanghai, China). The fluorescent probe 1-NPN (N-phenyl−1-naphthylamine, Macklin) was dissolved in methanol (Macklin) to a final stock concentration of 1 mM. To determine the binding constants between each protein and 1-NPN (K_1-NPN_), 1-NPN was added stepwise to 200 μL of the protein solution (2 μM) to achieve final concentrations ranging from 2 to 24 μM in increments of 2 μM. The mixture was gently agitated and incubated for 1 min at room temperature to reach equilibrium before recording the fluorescence intensity. This titration process continued until the binding reached saturation. Three independent repeats were performed for all measurements. The *K*_1-NPN_ values were then calculated by nonlinear regression using the Scatchard equation or a single-site binding model.

The dissociation constants (*K*_d_, representing *K*_1-NPN_) of the protein–probe complexes were determined by plotting the observed fluorescence intensity (*Y*) against the concentration of 1-NPN (*X*). The data were fitted using a one-site specific binding model based on the following equation:

$$Y=\frac{B_{max}\cdot X}{K_{d}+X}$$

where *B*_max_ represents the maximum fluorescence intensity. To further validate the binding stoichiometry and the reliability of the parameters, Scatchard plots were generated, where the ratio of bound/free 1-NPN was plotted against the concentration of bound 1-NPN to confirm a linear relationship characteristic of a single binding site.

For competitive binding assays, volatile compounds were diluted to 1 mM in methanol. A mixture of protein and 1-NPN (both at final concentrations of 2 μM) was added to the microplate and equilibrated for 1 min. Subsequently, each volatile ligand was added stepwise to achieve final concentrations ranging from 2 to 24 μM. After incubation for 2 min, fluorescence intensity was recorded. Each ligand was independently tested in triplicate. The *IC*_50_ values (the concentration of the competitor that resulted in a 50% reduction in the initial fluorescence of the protein−1-NPN complex) were calculated using the log (inhibitor) vs. response (three parameters) model by plotting the fluorescence intensity against the concentration of each volatile ligand. The dissociation constants (*K*_i_) of the competitors were then calculated based on the following equation [13]:

$$K_{i}={IC}_{50}/(1\;+\;[1\text{-}\mathrm{NPN}]/K_{1\text{-}NPN})$$

where [1-NPN] represents the concentration of free 1-NPN, and *K*_1-NPN_ denotes the dissociation constant of the DvirOBPs−1-NPN complex. The *K*_i_ value is inversely related to the binding affinity between the protein and the odorant ligand. Binding strength was classified according to *K*_i_ values as follows: *K*_i_ < 20 μmol/L indicates strong binding affinity, *K*_i_ > 50 μmol/L indicates no binding, and *K*_i_ values between 20 and 50 μmol/L indicate weak binding.

### 2.10. Statistical Analyses

All experimental data were presented as the mean ± standard error of the mean (SEM) from three independent biological replicates. Statistical analyses and curve fitting were performed using GraphPad Prism 9.5 (GraphPad Software, San Diego, CA, USA).

## 3. Results

### 3.1. Phylogenetic Analysis of the DvirOBP Family

**Figure 1:**
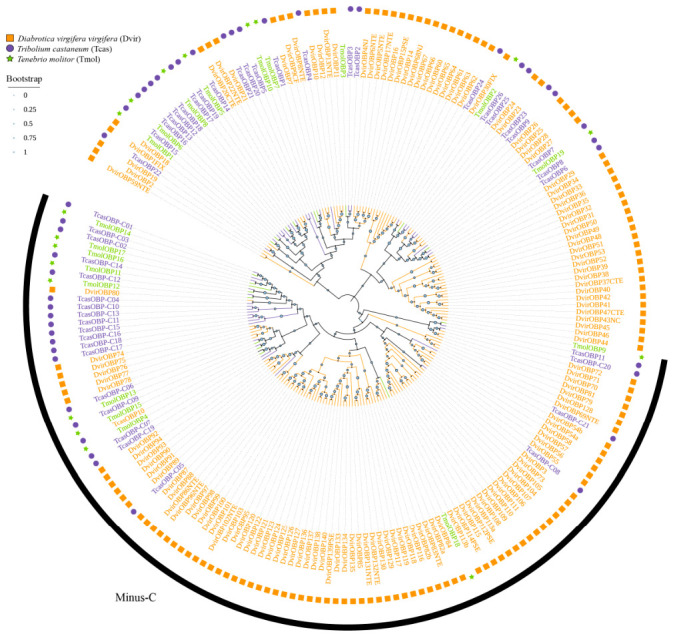
Circular neighbor-joining phylogenetic tree of the OBP family in *D. v. virgifera*. Sequences from *D. v. virgifera* (Dvir), *T. castaneum* (Tcas), and *T. molitor* (Tmol) are marked with orange squares, purple circles, and green pentagrams, respectively. Node support values were assessed using the bootstrap method with 1000 replicates.

Based on the previously published draft genome of *D. v. virgifera* [14], the OBP family can be divided into three groups: 50 classical OBPs (DvirOBP4 to 53), comprising 31 antennal binding protein II (ABPII) family members (DvirOBP23 to 53), and 90 Minus-C OBPs (DvirOBP54 to 140). Within this foundational dataset, specific structural variations were previously identified, including three alternatively spliced genes encoding two transcript isoforms each (DvirOBP54, DvirOBP82, and DvirOBP113), as well as one dimeric (DvirOBP107) and one tetrameric OBP gene (DvirOBP128). Due to strict quarantine regulations preventing the acquisition and laboratory rearing of live specimens for de novo sequencing, we utilized this comprehensive genomic data as the starting point for our study. Through homology-based screening, we targeted specific candidates for subsequent characterization. Notably, in our phylogenetic analysis, the selected target DvirOBP54b (a Minus-C OBP) forms a well-supported clade with DvirOBP54a (bootstrap support > 99%), suggesting that these two transcript isoforms may share similar functional characteristics (Figure 1).

### 3.2. Preliminary Virtual Screening and Specificity Analysis of the DvirOBP Family

To ensure the reliability of the 3D structures prior to molecular docking, the stereochemical quality and backbone conformation of the generated homology models were rigorously evaluated using Ramachandran plots. The evaluation results confirmed the high quality of the constructed models, providing a reliable structural basis for subsequent in silico evaluations (Supplementary Figures S1 and S2).

The binding potentials of 140 DvirOBP members with three target ligands, (E)-β-caryophyllene, 6-MBOA, and 2R,8R-MDP, were evaluated via family-wide molecular docking (Table 1). Data analysis showed a convergent distribution of binding energies (ΔG) for most members, clustering between −4.5 and −5.5 kcal/mol. Notably, DvirOBP54a exhibited a significantly superior theoretical affinity, particularly for (E)-β-caryophyllene (−7.89 kcal/mol). In contrast, DvirOBP54b showed a relatively average performance, with scores ranging from −5.2 to −5.3 kcal/mol across all three ligands, placing it near the family-wide mean.

**Figure 2:**
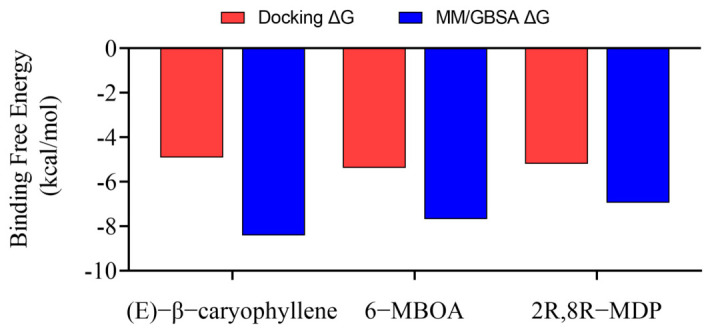
Comparison of binding free energy between DvirOBP54b and three target ligands. Red bars represent the binding free energies (ΔG) predicted by AutoDock Vina molecular docking, while blue bars represent the binding free energies (ΔG) calculated using the MM/GBSA method after molecular dynamics simulations. The three target ligands are (E)-β-caryophyllene, 6-MBOA, and 2R,8R-MDP.

Since molecular docking is based on static receptor conformations and employs simplified solvent effects, these scores may not fully represent true binding capabilities under physiological dynamic conditions. Given the representative phylogenetic position of DvirOBP54b, it was designated as a core candidate for further investigation. Molecular dynamics (MD) simulations and MM/GBSA calculations were subsequently employed for energetic refinement to assess binding specificity during dynamic conformational adjustments and to correct potential evaluation biases inherent in static docking. In contrast to the static docking results, a reversal in binding affinity ranking was observed after 50 ns MD simulations (Figure 2). (E)-β-caryophyllene exhibited the strongest refined binding affinity (ΔG = −8.42 kcal/mol), followed by 6-MBOA (ΔG = −7.68 kcal/mol), while 2R,8R-MDP showed the weakest (ΔG = −6.95 kcal/mol).

### 3.3. Family-Specific Analysis of Ligand Binding to DvirOBPs

**Figure 3:**
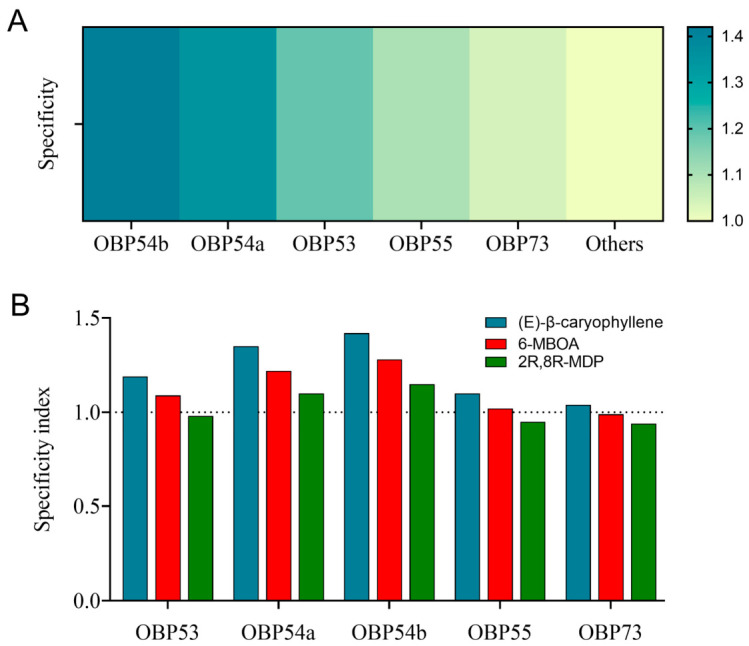
Pan-specificity screening and ranking of binding affinity across the DvirOBP family (*n* = 140). (**A**) Ranking of binding specificity index, highlighting the prominent selectivity of DvirOBP54b compared to other family members. The color gradient from yellow to blue indicates increasing specificity. (**B**) Binding specificity of candidate DvirOBPs against three key ligands: (E)-β-caryophyllene (blue), 6-MBOA (red), and 2R,8R-MDP (green). The dashed line represents the baseline specificity threshold.

Cross-family comparison (*n* = 140; Figure 3) revealed that DvirOBP54b exhibited the highest ligand-binding specificity after dynamic refinement, with specificity index values of 1.42 for (E)-β-caryophyllene, 1.28 for 6-MBOA, and 1.15 for 2R,8R-MDP. By comparison, DvirOBP54a displayed the second-highest binding specificity, yielding corresponding index values of 1.35, 1.22, and 1.10 for the respective ligands. These findings, integrated with its distinct phylogenetic status, confirm that DvirOBP54b occupies a high-specificity region within the DvirOBP family and plays a key role in mediating selective ligand recognition during olfactory processes.

This family-wide screening matrix provided the definitive comparative metrics for prioritizing DvirOBP54b over its close homolog, DvirOBP54a, for downstream characterization. Although preliminary molecular docking showed that DvirOBP54a yielded highly favorable absolute binding energies, the global specificity mapping across the three key ligands clearly demonstrated that DvirOBP54b exhibits a more prominent and pronounced selectivity advantage (Figure 3B). Given their exceptionally high sequence identity (>99%), proceeding with parallel, intensive downstream simulations for both duplicative paralogs would introduce severe data redundancy without delivering unique mechanistic insights. Since DvirOBP54b displayed the peak specificity profile within this cluster, it was uniquely prioritized as the sole representative target for all subsequent analyses, including productive long-term molecular dynamics simulations, MM/GBSA free energy profiling, and in vitro competitive binding validation.

### 3.4. Molecular Docking and Interaction Analysis of DvirOBP54b

**Figure 4:**
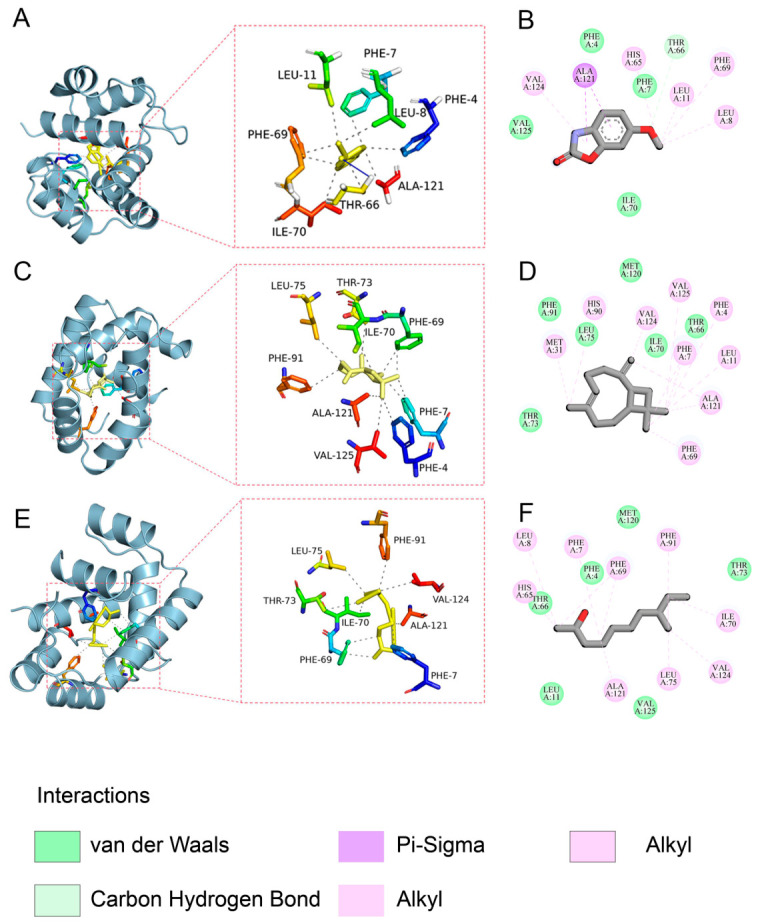
Binding mode of the ligands with the DvirOBP54b protein. The 3D (**A**) and 2D (**B**) binding mode of 6-MBOA with the DvirOBP54b protein; the 3D (**C**) and 2D (**D**) binding mode of (E)-β-caryophyllene with the DvirOBP54b protein; and the 3D (**E**) and 2D (**F**) binding mode of 2R,8R-MDP with the DvirOBP54b protein.

The 3D binding poses and 2D interaction networks of DvirOBP54b with three key ligands were analyzed using molecular docking (Figure 4).

For 6-MBOA, the ligand was positioned within the central hydrophobic cavity of DvirOBP54b (Figure 4A). As shown in the 2D interaction map (Figure 4B and Table S1), the complex was stabilized by a non-classical carbon–hydrogen bond (distance: 3.73 Å) between the carbonyl oxygen of 6-MBOA and the side chain of residue Thr66. Hydrophobic stabilization was predominantly provided by alkyl and π-alkyl interactions with residues Leu11, Phe69, Val124, Leu8, and His65 (with distances ranging from 3.30 Å to 3.74 Å). Additionally, a π-sigma interaction with Ala121 and van der Waals interactions with residues Phe4, Phe7, Val125, and Ile70 further contributed to the binding.

For (E)-β-caryophyllene, the ligand occupied the internal binding pocket primarily through nonpolar interactions (Figure 4C). The 2D interaction map (Figure 4D and Table S1) revealed that the binding was driven by alkyl and π-alkyl interactions with residues Met31, His90, Val124, Val125, Phe4, Phe7, Leu11, Ala121, and Phe69 (with distances ranging from 3.23 Å to 3.96 Å). Furthermore, an extensive network of van der Waals interactions involving residues Thr73, Phe91, Leu75, Ile70, Thr66 and Met120 was observed, which maintained the ligand in a stable orientation within the cavity.

For 2R,8R-MDP, the ligand adopted an extended conformation to fit the binding channel (Figure 4E). The 2D interaction network (Figure 4F and Table S1) showed that the binding was predominantly mediated by alkyl and π-alkyl interactions with residues His65, Leu8, Phe7, Phe69, Phe91, Ile70, Val124, Leu75, and Ala121 (with distances ranging from 3.62 Å to 3.99 Å). Furthermore, an extensive network of van der Waals interactions involving residues Leu11, Phe4, Thr66, Met120, Thr73, and Val125 was observed, which maintained the ligand in a stable orientation within the cavity. The interaction pattern was characterized by a broad distribution of hydrophobic contacts along the aliphatic chain and ester group of the molecule.

Further analysis revealed a structural “lock” mechanism driving stable ligand entrapment in DvirOBP54b (Figure S3). Specifically, coordinated intramolecular contacts between residue pairs on the α1 (Leu11–Phe7, Leu8–Phe4) and α4 (Thr66–Ile70) helices stabilize the boundary scaffolding. This internal lock restricts conformational fluctuations and effectively seals the hydrophobic cavity, minimizing ligand dissociation during olfactory recognition.

### 3.5. Conformational Dynamics of DvirOBP54b upon Ligand Binding

To gain a more dynamic understanding of the relative stability and binding interaction patterns of the three compounds with DvirOBP54b in a solvated environment, 50 ns molecular dynamics (MD) simulations were performed using NAMD 3.0 based on the docking poses. Binding free energies for each ligand were then calculated. In addition, simulations of the apo (ligand-free) protein were conducted under identical conditions to enable comparison with the protein–ligand complexes. The resulting trajectories were further analyzed using structural metrics (RMSD, RMSF) and interaction energy decomposition. The 50 ns MD simulations confirmed the structural stability of the complexes (Figure S4). The results indicated that the overall structure of DvirOBP54b remained stable upon binding with all three ligands (6-MBOA, (E)-β-caryophyllene, and 2R,8R-MDP). Among them, the (E)-β-caryophyllene complex exhibited the most stable RMSD trajectory (1.43 ± 0.06 nm), indicating the formation of a highly stable binding conformation. In contrast, the 2R,8R-MDP complex showed the largest fluctuations (1.87 ± 0.30 nm) (Figure S4A). Root mean square fluctuation (RMSF) analysis revealed that residues 14–20 (FCNAMPA) constitute a region of high flexibility within the protein, with a mean fluctuation value of 6.2 ± 0.8 Å. Notably, fluctuations in this region were significantly reduced in the (E)-β-caryophyllene complex (5.12 ± 0.58 Å), further supporting the enhanced stability of this complex (Figure S4B).

### 3.6. Cloning, Expression and Purification of DvirOBPs

**Figure 5:**
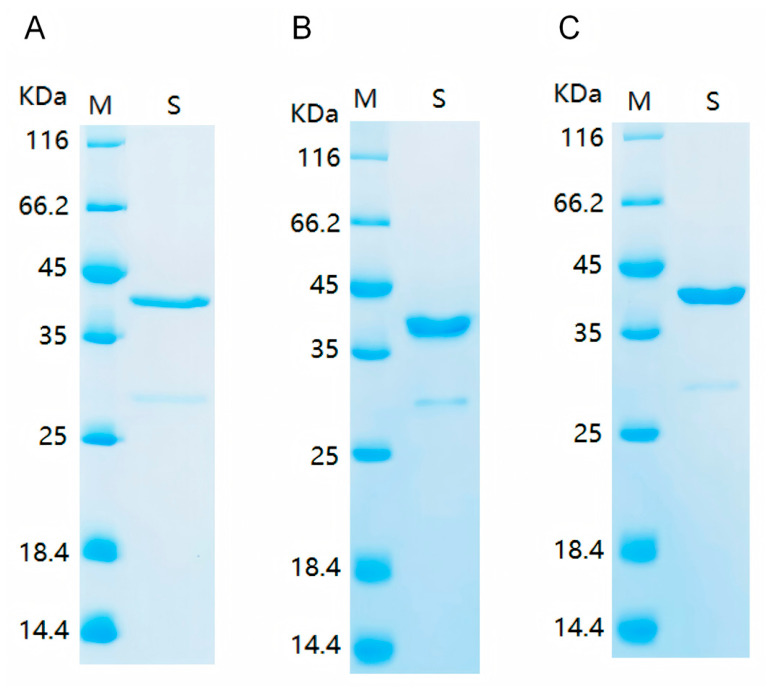
SDS-PAGE analysis of recombinant DvirOBPs. (**A**) DvirOBP53; (**B**) DvirOBP54b; (**C**) DvirOBP55. Lane M: protein molecular weight marker (kDa indicated on the left); Lane S: purified recombinant protein.

Based on a family-wide analysis of the ligand-binding specificities of DvirOBPs, three candidate proteins, namely DvirOBP53, DvirOBP54b, and DvirOBP55, were selected to investigate their interactions with the screened small-molecule ligands. The results showed that DvirOBP53 (Figure 5A), DvirOBP54b (Figure 5B), and DvirOBP55 (Figure 5C) were all successfully expressed with high purity. All three proteins exhibited clear single bands at approximately 35–45 kDa, consistent with their predicted molecular weights. While the native OBPs have predicted molecular weights of 15.1 kDa, 13.3 kDa, and 14.7 kDa, respectively, the recombinant fusion proteins exhibited clear single bands between the 35 kDa and 45 kDa markers. This accounts for the native proteins plus the N-terminal GST-tag (~26 kDa) and C-terminal His-tag, bringing the in silico-predicted molecular weights of the complete fusion constructs to approximately 40.9 to 42.8 kDa. The purified OBPs were then used for subsequent binding assays.

### 3.7. Fluorescence Competitive Binding Assays

The computational workflow, integrating homology modeling and family-wide molecular docking, served as a primary theoretical filter to explore binding potentials across the OBP repertoire. However, it is essential to acknowledge that in silico computational simulations can only represent real-world biological interactions to a certain extent. While docking results indicated a strong theoretical binding affinity for candidates like DvirOBP54a, these static predictions serve primarily as a comparative reference. To translate these theoretical indices into biological reality, empirical validation is indispensable. Next, representative proteins, including DvirOBP54b, DvirOBP53, and DvirOBP55, were preformed to conduct in vitro competitive fluorescence binding assays to accurately determine their actual physiological binding capabilities.

**Figure 6:**
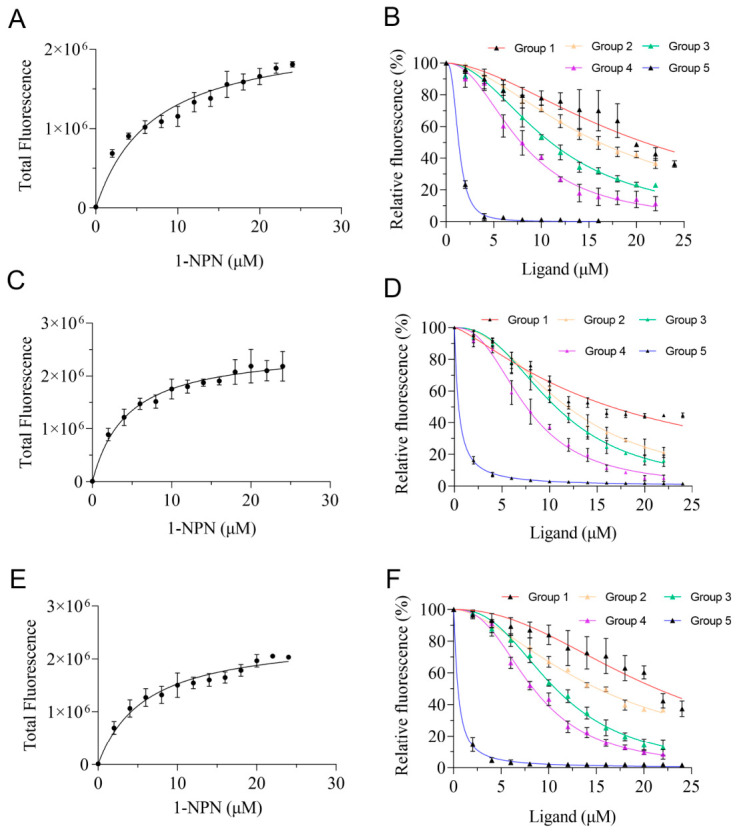
Competitive fluorescence ligand-binding assays of DvirOBPs to chemical ligands. The binding curves of 1-NPN to DvirOBP53 (**A**), DvirOBP54b (**C**), and DvirOBP55 (**E**). The competitive binding curves of DvirOBP53 (**B**), DvirOBP54b (**D**), and DvirOBP55 (**F**) with different mixtures of (E)-β-caryophyllene and 6-MBOA. The composition of the competing ligands in each group is as follows: Group 1, 100% (E)-β-caryophyllene; Group 2, 75% (E)-β-caryophyllene + 25% 6-MBOA; Group 3, 50% (E)-β-caryophyllene + 50% 6-MBOA; Group 4, 25% (E)-β-caryophyllene + 75% 6-MBOA; Group 5, 100% 6-MBOA.

Based on preliminary analyses, (E)-β-caryophyllene and 6-MBOA were selected as competitive ligands to evaluate their interactions with DvirOBPs using competitive fluorescence binding assays (Figure 6 and Figure S5 and Table 2). The results showed that the binding constants (*K*_1-NPN_) of DvirOBP53, DvirOBP54b, and DvirOBP55 with 1-NPN were 6.973 μM (Figure 6A), 4.408 μM (Figure 6C), and 5.959 μM (Figure 6E), respectively. Based on these values, the inhibition constants (*K*_i_) of the ligands toward DvirOBPs were further calculated. The competitive binding assays revealed that all three DvirOBPs exhibited a notably higher binding affinity for 6-MBOA compared to (E)-β-caryophyllene. Among the tested proteins, DvirOBP54b demonstrated the strongest overall binding capabilities, with *K*_i_ values of 0.34 µmol/L for 6-MBOA and 12.62 µmol/L for (E)-β-caryophyllene. DvirOBP55 showed the next highest affinities (*K*_i_ = 0.36 µmol/L for 6-MBOA; *K*_i_ = 16.91 µmol/L for (E)-β-caryophyllene), while DvirOBP53 displayed the weakest interactions (*K*_i_ = 1.07 µmol/L for 6-MBOA; *K*_i_ = 17.05 µmol/L for (E)-β-caryophyllene).

Furthermore, as 6-MBOA in the mixture increased, the *K*_i_ values steadily decreased, indicating progressively stronger binding affinities. For instance, with DvirOBP54b, the *K*_i_ values dropped from 9.07 µmol/L to 8.14 µmol/L and finally to 5.77 µmol/L as the 6-MBOA ratio increased from 25% to 50% and 75%, respectively. This consistent pattern was similarly observed for both DvirOBP53 and DvirOBP55. Overall, the experimental data confirm that these DvirOBPs possess a strong binding preference for the maize-derived volatile 6-MBOA, with DvirOBP54b exhibiting the most superior binding capability toward these key volatiles among its homologs.

## 4. Discussion

The western corn rootworm (*D. v. virgifera*) is a devastating pest, posing a significant threat to the global production of major gramineous crops such as maize and wheat [1,22]. The larvae damage maize by feeding on and tunneling into the root system, leading to plant lodging or even death [23]. In addition, *D. v. virgifera* can vector MCMV, potentially triggering large-scale viral outbreaks and causing severe yield losses [23]. Previous studies have shown that maize plants emit (E)-β-caryophyllene when attacked by western corn rootworm, a signal used by larvae to locate infested host plants [9]. Moreover, 6-MBOA, which is characterized by suitable volatility and water solubility, can effectively diffuse under varying soil moisture conditions and serves as a key attractant for larval host location, exhibiting synergistic effects with carbon dioxide [8]. The adult pheromone 2R,8R-MDP attracts males of the western corn rootworm and can interfere with mating [24]. The target ligands in this study were selected based on documented research confirming their critical biological roles in *D. v. virgifera* [8,9,10]. Specifically, 6-MBOA and (E)-β-caryophyllene serve as plant-derived volatile organic compounds (VOCs) that guide insects in host location, while 2R,8R-MDP functions as an insect sex pheromone. The high affinities of DvirOBPs for 2R,8R-MDP observed in this study provide a direct protein-level explanation for insect mating. Moreover, the binding profiles with (E)-β-caryophyllene and 6-MBOA further reflect the potential involvement of these proteins in foraging, establishing a clear connection between in vitro binding parameters and known ecological behaviors. In addition to these three key ligands, chemical recognition in the western corn rootworm involves a wide range of other compounds. Carbon dioxide acts as a universal attractant for larval host location [25,26]. Furthermore, specific maize root exudates, including indole, phenylpropanoids, and long-chain fatty acids, are also highly attractive to *D. v. virgifera* larvae [27,28]. Additionally, ethylene, a gaseous phytohormone regulating root growth, is utilized by second- and third-instar larvae as a reliable cue for selecting suitable roots [9]. Taken together, the diverse blend of sugars, aldehydes, and various terpenoids released from maize roots constitutes a dynamic chemical background essential for precise host recognition by this pest [29,30].

Odorant-binding proteins are small, soluble proteins present in the sensory organs of insects. Owing to their low molecular weight and the ease of recombinant expression and purification in vitro, OBPs have become important targets for elucidating the mechanisms of insect chemoreception, screening behavior-modulating compounds, and establishing environmentally friendly pest management strategies [31]. Structurally, OBPs typically adopt a globular fold composed predominantly of α-helices and usually contain three conserved disulfide bonds. Their core function is to recognize, bind, and transport odorant molecules, thereby participating in insect olfactory perception [32]. Despite the high evolutionary divergence among insect OBPs, this group of proteins shares several hallmark structural features, primarily defined by the conservation of their characteristic cysteine residues [33]. These structural motifs generally organize insect OBPs into Classic, Minus-C, Plus-C, Atypical, and Dimer subclasses [34]. Classic OBPs possess a typical six-cysteine motif that forms three interlocking disulfide bonds, rigidly stabilizing the α-helical scaffolding [35]. This six-cysteine pattern represents the most ancestral and frequently identified characteristic within every insect genome, encompassing major functional clades such as pheromone-binding proteins (PBPs) and general odorant-binding proteins (GOBPs) [36]. Conversely, Minus-C OBPs lack two specific cysteines (typically C_2_ and C_5_), resulting in a more flexible binding pocket sustained by only two disulfide bonds [37]. Plus-C OBPs feature additional cysteines (typically eight) along with a characteristic proline immediately following the sixth cysteine, significantly increasing structural complexity. Furthermore, atypical OBPs are distinguished by possessing 9–10 cysteines and an elongated C-terminus, while Dimer OBPs consist of two Classic OBP domains [36]. In the genome of *D. v. virgifera*, a diverse array of these structural subclasses has been identified (DvirOBPs). While their foundational structural classifications have been documented in previous genomic studies, the precise molecular mechanisms and dynamic forces governing their interactions with key host volatiles require further elucidation.

Extensive studies have been conducted on the identification and binding characterization of OBPs in various agriculturally insect species. For example, OBP2 from *Euplatypus parallelus* exhibits strong binding affinity to two host monoterpenes, myrcene and α-pinene, derived from *Hevea brasiliensis*, and also shows weak binding to the aggregation pheromones 2-methyl−3-buten−2-ol, (S)-(−)-cis-verbenol, and 2-phenyl−2-propanol [38]. The chemoreception of these semiochemicals directly mediates the beetle’s host-selection and aggregation behaviors. Similarly, OBP3, OBP5, and OBP6 from *Oides leucomelaena* display high binding affinity to β-caryophyllene among star anise volatiles [39], which is a critical step in driving their precise host-seeking behavior and adapting to plant defense mechanisms. Zhang et al. [40] demonstrated that OBP1 of *Monochamus alternatus* exhibits high affinity toward major host plant volatiles ((+)-α-pinene, β-pinene, β-caryophyllene, (+)-limonene, and verbenone) by using fluorescence competitive binding assays, suggesting that OBP–ligand interactions play a key role in host selection for insects.

To elucidate the olfactory recognition mechanism of the western corn rootworm, this study selected three representative volatile compounds as ligands based on key behavioral stages in its life cycle and performed molecular docking analyses with the corresponding OBPs. Molecular docking initially revealed that DvirOBP54a exhibited the highest binding affinity to (E)-β-caryophyllene, with the lowest average docking score among the three ligands (−7.3 kcal/mol), suggesting a broad binding spectrum toward multiple ligands. To further evaluate its potential as a key olfactory protein, ligand-binding specificity across the DvirOBP family was analyzed. The results showed that DvirOBP54b displayed the highest binding specificity, followed by DvirOBP54a. Given this difference, the structural basis underlying their functional divergence was investigated. Sequence alignment revealed a high degree of homology between the two proteins. However, within the key hydrophobic pocket motif (HEECKAQTGV), residue 52 differs: DvirOBP54b contains a phenylalanine (Phe52), whereas DvirOBP54a has a leucine (Leu52) at the corresponding position. This conserved aromatic substitution in DvirOBP54b potentially facilitates robust π–π stacking interactions with cyclic semiochemicals, thereby significantly sharpening its recognition accuracy over DvirOBP54a, which relies on weaker aliphatic van der Waals forces. Additionally, physicochemical analysis showed that the isoelectric point of DvirOBP54b (pI = 5.82) is lower than that of DvirOBP54a (*pI* = 6.45). Under physiological pH conditions (7.4), this difference may facilitate a more open conformation of the hydrophobic binding pocket in DvirOBP54b, thereby promoting ligand binding and release. Although DvirOBP54a exhibits broader ligand-binding capacity, DvirOBP54b demonstrates more pronounced ligand recognition characteristics in terms of binding specificity, key interacting residues, and favorable physicochemical properties. Therefore, DvirOBP54b, rather than its homolog DvirOBP54a, was ultimately selected as the representative target for subsequent in-depth analyses in this study.

Computational approaches, including homology modeling, molecular docking, and molecular dynamics (MD) simulations, are widely used in protein structure prediction and the elucidation of interaction mechanisms [41]. Several studies have demonstrated the effectiveness of integrating computational and experimental approaches to investigate OBP–ligand interactions. For instance, Li et al. [42] combined homology modeling, molecular docking, and fluorescence competitive binding assays to analyze the binding mechanism of OBP21 in *Dastarcus helophoroides*, revealing that while both hydrophobic interactions and hydrogen bonding contribute to ligand recognition, hydrophobic forces serve as the dominant driving factor. Similarly, OBP4 of *Tirathaba rufivena* was reported to exhibit strong binding affinity toward several host plant-derived compounds, including octyl methoxycinnamate, dibutyl phthalate, myristic acid, and palmitic acid [13]. In the present study, these computational tools were employed to investigate the binding modes and stability of DvirOBP54b in complex with three key chemical ligands. In contrast to classical insect OBPs that strictly possess six α-helices tightly regularized by three disulfide bonds, computational structural analysis of DvirOBP54b revealed a distinctive seven-α-helix topology. As a characteristic Minus-C OBP, DvirOBP54b harbors only four conserved cysteine residues, which cross-link to form two intra-chain disulfide bridges. This missing pair of disulfide bonds inherently compromises the rigidity of the core scaffolding, generating pronounced conformational flexibility [43]. Crucially, such structural plasticity is postulated to endow the unique C-terminal seventh α-helix with heightened dynamic adaptability during protein–ligand accommodation. Insect OBPs that simultaneously feature this reduced disulfide network and an extra C-terminal α-helix are increasingly recognized to exhibit broader ligand-binding spectra and superior spatial tolerance toward diverse small-molecule ligands. This flexible, non-rigid scanning paradigm likely represents a pivotal evolutionary adaptation allowing the insect olfactory machinery to accurately scan and perceive highly variable and dynamic blends of plant-derived volatile semiochemicals. This structural paradigm directly aligns with AmalOBP8 from *Agrilus mali*, a homologous coleopteran Minus-C OBP featuring an extra C-terminal α-helix and a reduced disulfide network. Molecular dynamics and functional analyses of AmalOBP8 confirmed that this heightened flexibility, coupled with a dense network of conserved hydrophobic residues, enables the protein to dynamically adapt and expand its binding spectrum toward diverse plant volatiles. Intriguingly, our structural topology analysis revealed that the intrinsic architectural rigidity of DvirOBP54b plays a decisive role in ligand trapping, driven by specific intra-protein interaction networks within its helical scaffolding [44]. Specifically, residue pairs Leu11–Phe7 and Leu8–Phe4 on the α1 helix, alongside the Thr66–Ile70 pair on the α4 helix, establish highly coordinated intramolecular contacts. These localized networks function synergistically as a structural “lock” that stabilizes the spatial orientation of the boundary helices. By restricting global conformational fluctuations, this internal locking matrix effectively seals the hydrophobic cavity once a volatile semiochemical enters, thereby minimizing ligand dissociation and ensuring robust chemical recognition during olfactory perception. Our results consistently indicated that hydrophobic interactions dominate the binding process, with a hydrophobic pocket formed by residues Phe7, Phe69, Ile70, and Ala121 playing a central role in determining binding specificity. These residues contributed substantially to the interactions with all three odorant molecules. In insect OBPs, the internal binding cavity is characteristically lined with a high proportion of hydrophobic and aromatic amino acids, which are essential for capturing and accommodating volatile, nonpolar ligands [45]. Specifically, the aromatic rings of phenylalanine (Phe7 and Phe69) frequently act as key structural anchors, establishing stable π-alkyl, π-sigma, or van der Waals interactions with the carbon skeletons of the odorants [38,46]. Concurrently, aliphatic residues such as Isoleucine (Ile70) and Alanine (Ala121) contribute to the overall hydrophobicity and spatial geometry of the pocket, effectively shielding the lipophilic ligands from the surrounding aqueous environment [47]. The critical functional roles of these specific amino acid types are strongly supported by structural studies on other coleopteran OBPs. For example, highly conserved hydrophobic residues (particularly Phe and Ile) in the binding pocket of OBP2 from *E. parallelus* are the primary contributors to the selective recognition of host-derived terpenes via extensive alkyl and π-alkyl interactions [38]. Therefore, the synergistic contribution of these aromatic and aliphatic residues ensures that DvirOBP54b can efficiently bind and transport poorly water-soluble semiochemicals in complex physiological contexts. Notably, for 6-MBOA, an additional specific hydrogen bond involving Thr66 was observed, which likely enhances local binding stability. However, this ligand did not exhibit the strongest overall binding affinity, suggesting that a synergistic network of hydrophobic, van der Waals, and electrostatic interactions governs the overall OBP–ligand binding affinity.

Importantly, in natural foraging environments, insects rarely encounter these plant volatiles in isolation. Instead, they must parse complex, multi-component odor plumes where molecules like 6-MBOA and (E)-β-caryophyllene persistently co-exist. To address this real-world scenario, our experimental data evaluating mixed-ligand interactions provides vital physiological context. When these two molecules were presented simultaneously, we observed a clear modulatory effect on the binding dynamics across the tested OBPs. Specifically, as the proportion of the highly preferred 6-MBOA increased in the mixture, the overall dissociation constants (*K*_i_) for all three DvirOBPs steadily decreased, indicating progressively stronger complex formation. This suggests that in a natural blend, the presence of a primary high-affinity ligand (like 6-MBOA) strongly dictates the overall binding efficiency, while secondary ligands ((E)-β-caryophyllene) can still be accommodated within the protein matrix. When comparing the homologs within this realistic context, while DvirOBP55 serves as a highly effective broad-spectrum binder, DvirOBP54b demonstrates an even stronger real-world binding capability for (E)-β-caryophyllene (*K*_i_ = 12.62 µmol/L) alongside its high affinity for 6-MBOA. This reinforces the hypothesis that DvirOBP54b has evolved a functionally optimized binding pocket specifically tailored to this critical maize-derived foraging blend. Ultimately, rather than operating under a mechanism of strict ligand exclusivity, our mixed-binding data reveals a clear pattern of overlapping and interactive ligand responsiveness. In this combinatorial model, complex natural odorant blends are detected by multiple OBPs with varying, yet overlapping, sensitivities. Such functional redundancy is crucial for the insect olfactory system, significantly expanding its discriminatory power and coding capacity, thereby allowing the insect to reliably navigate complex environmental cues using a limited repertoire of binding proteins.

Furthermore, the stability of hydrogen bonds is highly dependent on their geometric configuration and the physicochemical properties of the local microenvironment. From a structural perspective, OBPs function as transporters of lipophilic odorant molecules and typically possess highly hydrophobic binding cavities. In contrast, ligands capable of forming hydrogen bonds often exhibit higher polarity, which may increase their partitioning tendency toward aqueous environments, thereby partially reducing their stability within hydrophobic pockets [48,49,50]. This theoretical framework aligns perfectly with our MD-based binding free energy calculations, which identified (E)-β-caryophyllene as having the strongest binding affinity under dynamic physiological conditions. This superiority is consistent with its extensive hydrophobic contacts within the binding cavity, facilitating stable retention within the pocket [37]. Although 6-MBOA and 2R,8R-MDP exhibited a comparable number of hydrophobic interactions, 6-MBOA displayed a higher binding affinity than the latter, likely owing to the supplementary stabilization provided by its specific hydrogen bond. This observed structure–activity relationship is consistent with previous findings reported by Bourdon et al. [51].

## 5. Conclusions

Insect OBPs generally possess the ability to recognize groups of structurally similar odorant molecules. Their inherent structural stability, reversible binding properties, and ease of heterologous expression make them promising candidates for applications in electrochemical sensing. In this study, the molecular mechanisms underlying the olfactory recognition of key chemical ligands were elucidated in WCR, through an integrated approach involving phylogenetic analysis, AlphaFold2-based protein structure prediction, molecular docking, and MD simulations, complemented by in vitro fluorescence competitive binding assays. Our results indicated that within the expansive OBP family of the WCR, DvirOBP54b exhibits exceptionally high ligand-binding specificity and demonstrates significant affinity for host-derived 6-MBOA and (E)-β-caryophyllene. Structural analysis further reveals that the binding of DvirOBP54b to these ligands is primarily driven by hydrophobic interactions involving key residues such as Phe7, Phe69, Ile70, and Ala121, while a specific hydrogen bond formed with Thr66 provides additional structural stability for the recognition of 6-MBOA. MD simulations confirmed the high stability of these protein–ligand complexes under physiological dynamic conditions, and the experimentally determined dissociation constants (*K*_i_) were highly consistent with the computational simulation results, fully demonstrating the core role of DvirOBP54b in WCR olfactory recognition. This research not only reveals the coding mechanisms of WCR for host odor molecules at the molecular level, but also provides critical molecular targets and a theoretical basis for the development of high-efficiency OBP-based attractants, olfactory disruptors, and novel biosensors, which hold significant importance for advancing green and precise management strategies against this major quarantine pest.

## Figures and Tables

**Table 1:** Binding affinities (kcal/mol) of three target ligands docked into the binding pockets of DvirOBPs.

OBP Classes	Gene Ligand	(E)-β- Caryophyllene	6- MBOA	2R,8R -MDP	Specificity Index	OBP Classes	Gene Ligand	(E)-β- Caryophyllene	6- MBOA	2R,8R -MDP	Specificity Index	OBP Classes	Gene Ligand	(E)-β- Caryophyllene	6- MBOA	2R,8R -MDP	Specificity Index
Plus-C	OBP1	−4.85	−5.12	−4.67	0.95	ABPII	OBP47	−5.23	−5.45	−5.12	1.02	Minus-C	OBP93	−5.01	−5.34	−4.98	0.99
Plus-C	OBP2	−5.03	−5.28	−4.89	0.98	ABPII	OBP48	−5.01	−5.23	−4.98	0.98	Minus-C	OBP94	−5.12	−5.45	−5.01	1.01
Plus-C	OBP3	−4.21	−4.56	−4.03	0.83	ABPII	OBP49	−4.98	−5.12	−4.89	0.97	Minus-C	OBP95	−5.23	−5.56	−5.12	1.03
Plus-C	OBP107	−5.23	−5.45	−5.12	1.02	ABPII	OBP50	−4.98	−5.12	−4.89	0.97	Minus-C	OBP96	−4.45	−4.67	−4.34	0.87
Plus-C	OBP128	−4.34	−4.56	−4.23	0.85	ABPII	OBP51	−5.34	−5.56	−5.23	1.04	Minus-C	OBP97	−5.34	−5.56	−5.23	1.04
Classic	OBP4	−5.34	−5.67	−5.12	1.04	ABPII	OBP52	−5.23	−5.45	−5.12	1.02	Minus-C	OBP98	−5.45	−5.67	−5.34	1.07
Classic	OBP5	−5.45	−5.78	−5.23	1.07	ABPII	OBP53	−6.12	−5.89	−5.67	1.14	Minus-C	OBP99	−5.56	−5.78	−5.45	1.09
Classic	OBP6	−5.56	−5.89	−5.34	1.09	Minus-C	OBP54a	−7.89	−7.23	−6.78	1.35	Minus-C	OBP100	−5.67	−5.89	−5.56	1.11
Classic	OBP7	−4.67	−4.89	−4.56	0.91	Minus-C	OBP54b	−8.42	−7.68	−6.95	1.42	Minus-C	OBP101	−5.78	−6.01	−5.67	1.13
Classic	OBP8	−4.98	−5.23	−4.89	0.98	Minus-C	OBP55	−6.45	−6.12	−5.89	1.22	Minus-C	OBP102	−4.89	−5.12	−4.78	0.96
Classic	OBP9	−5.12	−5.34	−5.01	1.00	Minus-C	OBP56	−5.78	−6.01	−5.56	1.12	Minus-C	OBP103	−5.01	−5.23	−4.98	0.98
Classic	OBP10	−5.67	−5.89	−5.45	1.10	Minus-C	OBP57	−5.45	−5.67	−5.34	1.07	Minus-C	OBP104	−4.78	−5.01	−4.67	0.94
Classic	OBP11	−5.23	−5.45	−5.12	1.02	Minus-C	OBP58	−5.34	−5.56	−5.23	1.04	Minus-C	OBP105	−4.67	−4.89	−4.56	0.91
Classic	OBP12	−5.01	−5.23	−4.98	0.98	Minus-C	OBP59	−4.89	−5.12	−4.78	0.96	Minus-C	OBP106	−5.12	−5.34	−5.01	1.00
Classic	OBP13	−5.34	−5.56	−5.23	1.04	Minus-C	OBP60	−5.01	−5.23	−4.98	0.98	Minus-C	OBP108	−4.98	−5.12	−4.89	0.97
Classic	OBP14	−5.78	−6.01	−5.56	1.12	Minus-C	OBP61	−5.12	−5.34	−5.01	1.00	Minus-C	OBP109	−5.01	−5.23	−4.98	0.98
Classic	OBP15	−5.45	−5.67	−5.34	1.07	Minus-C	OBP62	−5.23	−5.45	−5.12	1.02	Minus-C	OBP110	−5.12	−5.34	−5.01	1.00
Classic	OBP16	−4.89	−5.12	−4.78	0.96	Minus-C	OBP63	−5.34	−5.56	−5.23	1.04	Minus-C	OBP111	−5.23	−5.45	−5.12	1.02
Classic	OBP17	−5.23	−5.45	−5.01	1.02	Minus-C	OBP64	−5.45	−5.67	−5.34	1.07	Minus-C	OBP112	−4.89	−5.12	−4.78	0.96
Classic	OBP18	−4.45	−4.67	−4.34	0.87	Minus-C	OBP65	−5.56	−5.78	−5.45	1.09	Minus-C	OBP113a	−5.01	−5.23	−4.98	0.98
Classic	OBP19	−5.56	−5.78	−5.34	1.08	Minus-C	OBP66	−4.78	−5.01	−4.67	0.94	Minus-C	OBP113b	−5.12	−5.34	−5.01	1.00
Classic	OBP20	−5.01	−5.23	−4.98	0.98	Minus-C	OBP67	−4.67	−4.89	−4.56	0.91	Minus-C	OBP114	−5.23	−5.45	−5.12	1.02
Classic	OBP21	−4.98	−5.12	−4.89	0.97	Minus-C	OBP68	−4.56	−4.78	−4.45	0.89	Minus-C	OBP115	−5.34	−5.56	−5.23	1.04
Classic	OBP22	−5.12	−5.34	−5.01	1.00	Minus-C	OBP69	−4.45	−4.67	−4.34	0.87	Minus-C	OBP116	−4.78	−5.01	−4.67	0.94
ABPII	OBP23	−4.67	−4.89	−4.56	0.91	Minus-C	OBP70	−5.01	−5.23	−4.98	0.98	Minus-C	OBP117	−4.67	−4.89	−4.56	0.91
ABPII	OBP24	−4.78	−5.01	−4.67	0.94	Minus-C	OBP71	−5.12	−5.34	−5.01	1.00	Minus-C	OBP118	−4.56	−4.78	−4.45	0.89
ABPII	OBP25	−5.12	−5.34	−5.01	1.00	Minus-C	OBP72	−5.23	−5.45	−5.12	1.02	Minus-C	OBP119	−4.45	−4.67	−4.34	0.87
ABPII	OBP26	−5.23	−5.45	−5.12	1.02	Minus-C	OBP73	−5.78	−6.01	−5.56	1.12	Minus-C	OBP120	−5.01	−5.23	−4.98	0.98
ABPII	OBP27	−4.98	−5.12	−4.89	0.97	Minus-C	OBP74	−4.98	−5.12	−4.89	0.97	Minus-C	OBP121	−5.12	−5.34	−5.01	1.00
ABPII	OBP28	−5.23	−5.45	−5.12	1.02	Minus-C	OBP75	−5.01	−5.23	−4.98	0.98	Minus-C	OBP122	−5.23	−5.45	−5.12	1.02
ABPII	OBP29	−5.34	−5.56	−5.23	1.04	Minus-C	OBP76	−4.89	−5.12	−4.78	0.96	Minus-C	OBP123	−5.34	−5.56	−5.23	1.04
ABPII	OBP30	−5.67	−5.89	−5.45	1.10	Minus-C	OBP77	−5.12	−5.34	−5.01	1.00	Minus-C	OBP124	−4.89	−5.12	−4.78	0.96
ABPII	OBP31	−5.23	−5.45	−5.12	1.02	Minus-C	OBP78	−5.23	−5.45	−5.12	1.02	Minus-C	OBP125	−5.01	−5.23	−4.98	0.98
ABPII	OBP32	−5.01	−5.23	−4.98	0.98	Minus-C	OBP79	−5.34	−5.56	−5.23	1.04	Minus-C	OBP126	−5.12	−5.34	−5.01	1.00
ABPII	OBP33	−4.33	−4.56	−4.23	0.85	Minus-C	OBP80	−5.45	−5.67	−5.34	1.07	Minus-C	OBP127	−5.23	−5.45	−5.12	1.02
ABPII	OBP34	−4.45	−4.67	−4.34	0.87	Minus-C	OBP81	−4.67	−4.89	−4.56	0.91	Minus-C	OBP129	−5.45	−5.67	−5.34	1.07
ABPII	OBP35	−5.34	−5.56	−5.23	1.04	Minus-C	OBP82a	−5.01	−5.23	−4.98	0.98	Minus-C	OBP130	−5.56	−5.78	−5.45	1.09
ABPII	OBP36	−5.23	−5.45	−5.12	1.02	Minus-C	OBP82b	−5.12	−5.34	−5.01	1.00	Minus-C	OBP131	−4.78	−5.01	−4.67	0.94
ABPII	OBP37	−5.12	−5.34	−5.01	1.00	Minus-C	OBP83	−5.23	−5.45	−5.12	1.02	Minus-C	OBP132	−4.67	−4.89	−4.56	0.91
ABPII	OBP38	−5.01	−5.23	−4.98	0.98	Minus-C	OBP84	−5.34	−5.56	−5.23	1.04	Minus-C	OBP133	−5.01	−5.23	−4.98	0.98
ABPII	OBP39	−4.98	−5.12	−4.89	0.97	Minus-C	OBP85	−5.45	−5.67	−5.34	1.07	Minus-C	OBP134	−5.12	−5.34	−5.01	1.00
ABPII	OBP40	−5.34	−5.56	−5.23	1.04	Minus-C	OBP86	−4.34	−4.56	−4.23	0.85	Minus-C	OBP135	−5.23	−5.45	−5.12	1.02
ABPII	OBP41	−5.67	−5.89	−5.45	1.10	Minus-C	OBP87	−4.45	−4.67	−4.34	0.87	Minus-C	OBP136	−4.89	−5.12	−4.78	0.96
ABPII	OBP42	−5.78	−6.01	−5.56	1.12	Minus-C	OBP88	−4.56	−4.78	−4.45	0.89	Minus-C	OBP137	−4.78	−5.01	−4.67	0.94
ABPII	OBP43	−5.23	−5.45	−5.12	1.02	Minus-C	OBP89	−4.67	−4.89	−4.56	0.91	Minus-C	OBP138	−4.67	−4.89	−4.56	0.91
ABPII	OBP44	−5.34	−5.56	−5.23	1.04	Minus-C	OBP90	−4.78	−5.01	−4.67	0.94	Minus-C	OBP139	−4.56	−4.78	−4.45	0.89
ABPII	OBP45	−5.67	−5.89	−5.45	1.10	Minus-C	OBP91	−4.89	−5.12	−4.78	0.96	Minus-C	OBP140	−4.33	−4.67	−4.12	0.85
ABPII	OBP46	−5.78	−6.01	−5.56	1.12	Minus-C	OBP92	−4.98	−5.23	−4.89	0.98						

**Table 2:** Binding affinities of different ligands to DvirOBPs.

OBPs K_i_ µmol/L	(E)-β-Caryophyllene	(E)-β-Caryophyllene (75%) 6-MBOA (25%)	(E)-β-Caryophyllene (50%) 6-MBOA (50%)	(E)-β-Caryophyllene (25%) 6-MBOA (75%)	6-MBOA
DvirOBP53	17.05	13.17	8.98	6.42	1.07
DvirOBP54b	12.62	9.07	8.14	5.77	0.34
DvirOBP55	16.91	12.00	8.48	6.63	0.36

## Data Availability

The data presented in this study are available on request from the corresponding authors.

## Supplementary Materials

The following supporting information can be downloaded at: https://www.mdpi.com/article/10.3390/insects17060595/s1, Figure S1: Homology models of representative *D. v. virgifera* odorant-binding proteins; Figure S2: Ramachandran plot validation for the structural models of four representative DvirOBP subclasses; Figure S3: Local intramolecular stabilization networks within the DvirOBP54b pocket; Figure S4: Results of the 50 ns Molecular Dynamics Simulation; Figure S5: Scatchard plot analysis of 1-NPN binding to three representative DvirOBPs; Table S1: Intramolecular interactions of DvirOBP54b with 6-MBOA, (E)-β-caryophyllene, and 2R,8R-MDP.

## Abbreviations

The following abbreviations are used in this manuscript:

WCR	western corn rootworm
OBPs	odorant-binding proteins
VOCs	volatile organic compounds
6-MBOA	6-methoxy−2-benzoxazolinone
2R,8R-MDP	8R-methyl−2R-decyl propanoate
MD	molecular dynamics
1-NPN	N-phenyl−1-naphthylamine

## References

[1] Fishilevich E., Vélez A.M., Storer N.P., Li H., Bowling A.J., Rangasamy M., Worden S.E., Narva K.E., Siegfried B.D. (2016). RNAi as a management tool for the western corn rootworm, *Diabrotica virgifera virgifera*. Pest Manag. Sci..

[2] Ferracini C., Blandino M., Rigamonti I.E., Jucker C., Busato E., Saladini M.A., Reyneri A., Alma A. (2021). Chemical-based strategies to control the western corn rootworm, *Diabrotica virgifera virgifera* LeConte. Crop Prot..

[3] Mitchell P. Costs and benefits of controlling pest *Diabrotica* in maize in the United States. Proceedings of the 24th IWG Conference.

[4] Schumann M., Ladin Z.S., Beatens J., Hiltpold I. (2018). Navigating on a chemical radar: Usage of root exudates by foraging *Diabrotica virgifera virgifera* larvae. J. Appl. Entomol..

[5] Paddock K.J., Corcoran J.A. (2025). Life-stage dependent behavior mimics chemosensory repertoire diversity in a belowground, specialist herbivore. G3 Genes Genomes Genet..

[6] Hiltpold I., Hibbard B.E. (2016). Neonate larvae of the specialist herbivore *Diabrotica virgifera virgifera* do not exploit the defensive volatile (E)-β-caryophyllene in locating maize roots. J. Pest Sci..

[7] Hibbard B., Bjostad L. (1988). Behavioral responses of western corn rootworm larvae to volatile semiochemicals from corn seedlings. J. Chem. Ecol..

[8] Bjostad L.B., Hibbard B.E. (1992). 6-Methoxy-2-benzoxazolinone: A semiochemical for host location by western corn rootworm larvae. J. Chem. Ecol..

[9] Robert C.A., Erb M., Duployer M., Zwahlen C., Doyen G.R., Turlings T.C. (2012). Herbivore-induced plant volatiles mediate host selection by a root herbivore. New Phytol..

[10] Guss P., Sonnet P., Carney R., Branson T., Tumlinson J. (1984). Response of *Diabrotica virgifera virgifera*, *D. v. zeae*, and *D. porracea* to stereoisomers of 8-methyl-2-decyl propanoate. J. Chem. Ecol..

[11] Qian Q., Guo X., Wu L., Cui J., Gao H., Yang Y., Xu H., Lu Z., Zhu P. (2024). Molecular characterization of plant volatile compound interactions with *Cnaphalocrocis medinalis* odorant-binding proteins. Plants.

[12] Śledź P., Caflisch A. (2018). Protein structure-based drug design: From docking to molecular dynamics. Curr. Opin. Struct. Biol..

[13] Zhou X., Wang Z., Cui G., Du Z., Qian Y., Yang S., Liu M., Guo J. (2022). Binding properties of odorant-binding protein 4 of *Tirathaba rufivena* to *Areca catechu* volatiles. Plants.

[14] Coates B.S., Walden K.K., Lata D., Vellichirammal N.N., Mitchell R.F., Andersson M.N., McKay R., Lorenzen M.D., Grubbs N., Wang Y.H. (2023). A draft *Diabrotica virgifera virgifera* genome: Insights into control and host plant adaption by a major maize pest insect. BMC Genom..

[15] Liu S., Rao X.J., Li M.Y., Feng M.F., He M.Z., Li S.G. (2015). Identification of candidate chemosensory genes in the antennal transcriptome of *Tenebrio molitor* (Coleoptera: Tenebrionidae). Comp. Biochem. Physiol. Part D. Genom. Proteom..

[16] Kim S., Chen J., Cheng T., Gindulyte A., He J., He S., Li Q., Shoemaker B.A., Thiessen P.A., Yu B. (2023). PubChem 2023 update. Nucleic Acids Res..

[17] O’Boyle N.M., Banck M., James C.A., Morley C., Vandermeersch T., Hutchison G.R. (2011). Open Babel: An open chemical toolbox. J. Cheminform..

[18] Jumper J., Evans R., Pritzel A., Green T., Figurnov M., Ronneberger O., Tunyasuvunakool K., Bates R., Žídek A., Potapenko A. (2021). Highly accurate protein structure prediction with AlphaFold. Nature.

[19] Mirdita M., Schütze K., Moriwaki Y., Heo L., Ovchinnikov S., Steinegger M. (2022). ColabFold: Making protein folding accessible to all. Nat. Methods.

[20] Wang R., Duan L., Zhao B., Zheng Y., Chen L. (2024). Molecular recognition between volatile molecules and odorant binding proteins 7 by homology modeling, molecular docking and molecular dynamics simulation. J. Sci. Food Agric..

[21] Wang L., Hou M., Liang C., Xu Q., Lu Y., Zhao Z. (2024). Role of odorant binding protein C12 in the response of *Tribolium castaneum* to chemical agents. Pestic. Biochem. Physiol..

[22] Meinke L.J., Sappington T.W., Onstad D.W., Guillemaud T., Miller N.J., Komáromi J., Levay N., Furlan L., Kiss J., Toth F. (2009). Western corn rootworm (*Diabrotica virgifera virgifera* LeConte) population dynamics. Agric. For. Entomol..

[23] Bažok R., Lemić D., Chiarini F., Furlan L. (2021). Western corn rootworm (*Diabrotica virgifera virgifera* LeConte) in Europe: Current status and sustainable pest management. Insects.

[24] Krysan J., Wilkin P., Tumlinson J., Sonnet P., Carney R., Guss P. (1986). Responses of *Diabrotica lemniscata* and *D. longicornis* (Coleoptera: Chrysomelidae) to stereoisomers of 8-methyl-2-decyl-propanoate and studies on the pheromone of *D. longicornis*. Ann. Entomol. Soc. Am..

[25] Anitha V., Kalasariya R., Shudeer S.S.K., Ramya R. (2024). Impacts of increasing atmospheric carbon dioxide on insect pests and natural enemies. Int. J. Adv. Biochem. Res..

[26] Bede J.C., Blande J.D. (2025). Effects of elevated CO_2_ and O_3_ on aboveground brassicaceous plant–insect interactions. Annu. Rev. Entomol..

[27] Zhu L., Yang H., Li P., Dong L., Zhao S., Lv H., Crickmore N., Zhou X., Zhang Y., Guo Z. (2026). Plant strategies against herbivorous insects. J. Integr. Plant Biol..

[28] Bernklau E., Hibbard B., Bjostad L. (2016). Toxic and behavioural effects of free fatty acids on western corn rootworm (Coleoptera: Chrysomelidae) larvae. J. Appl. Entomol..

[29] Hinsinger P., Gobran G.R., Gregory P.J., Wenzel W.W. (2005). Rhizosphere geometry and heterogeneity arising from root-mediated physical and chemical processes. New Phytol..

[30] Hiltpold I., Bernklau E., Bjostad L.B., Alvarez N., Miller-Struttmann N.E., Lundgren J.G., Hibbard B.E. (2013). Nature, evolution and characterisation of rhizospheric chemical exudates affecting root herbivores. Adv. Insect Physiol..

[31] Rana A., Sharma D., Choudhary K., Kumari P., Ruchika K., Yangchan J., Kumar S. (2024). Insight into insect odorant binding proteins: An alternative approach for pest management. J. Nat. Pestic. Res..

[32] Tzotzos G. (2022). A comparative evaluation of the structural and dynamic properties of insect odorant binding proteins. Biomolecules.

[33] Wang Q., Shentu X., Yu X., Liu Y. (2025). Insect Odorant-binding proteins (OBPs) and chemosensory proteins (CSPs): Mechanisms and research perspectives in mediating insecticide resistance. Biology.

[34] Leal W.S. (2025). Odorant reception in insects: Functional and evolutionary perspectives. Annu. Rev. Entomol..

[35] Gouda M., Subramanian S. (2026). Odorant-binding proteins and chemosensory proteins in insects: Structural insights, functional plasticity, and prospects for targeted pest management. Mol. Biol. Rep..

[36] Zhou J.-J. (2010). Odorant-binding proteins in insects. Vitam. Horm..

[37] Liu L., Li Y., Yang H., Wang F., Huang Q. (2025). Molecular characterization of a Minus-C odorant-binding protein from *Cyrtotrachelus buqueti* (Coleoptera: Curculionidae). Front. Physiol..

[38] Cui G., Zhou X., Wang Q., Zhang K., Qin L., Guo J. (2023). The sequence characteristics and binding properties of the odorant-binding protein 2 of *Euplatypus parallelus* to semiochemicals. Int. J. Mol. Sci..

[39] Zhao N., Li K., Ma H., Hu L., Yang Y., Liu L. (2024). Molecular characterization of odorant-binding protein genes associated with host-seeking behavior in *Oides leucomelaena*. Int. J. Mol. Sci..

[40] Zhang F., Merchant A., Zhao Z., Zhang Y., Zhang J., Zhang Q., Wang Q., Zhou X., Li X. (2020). Characterization of MaltOBP1, a Minus-C odorant-binding protein, from the Japanese pine sawyer beetle, *Monochamus alternatus* Hope (Coleoptera: Cerambycidae). Front. Physiol..

[41] Schmidt T., Bergner A., Schwede T. (2014). Modelling three-dimensional protein structures for applications in drug design. Drug Discov. Today.

[42] Li D.Z., Yu G.Q., Yi S.C., Zhang Y., Kong D.X., Wang M.Q. (2015). Structure-based analysis of the ligand-binding mechanism for DhelOBP21, a C-minus odorant binding protein, from *Dastarcus helophoroides* (Fairmaire; Coleoptera: Bothrideridae). Int. J. Biol. Sci..

[43] Zheng Z.-C., Li D.-Z., Zhou A., Yi S.-C., Liu H., Wang M.-Q. (2016). Predicted structure of a Minus-C OBP from *Batocera horsfieldi* (Hope) suggests an intermediate structure in evolution of OBPs. Sci. Rep..

[44] Li D., Li C., Liu D. (2021). Analyses of structural dynamics revealed flexible binding mechanism for the *Agrilus mali* odorant binding protein 8 towards plant volatiles. Pest Manag. Sci..

[45] Abendroth J.A., Moural T.W., Wei H., Zhu F. (2023). Roles of insect odorant binding proteins in communication and xenobiotic adaptation. Front. Insect Sci..

[46] Peng P., Tang Y., Yang S., Mo Y., Huang G., Chen Y. (2025). Odorant-binding protein 84a-1 mediates detection of Guire No. 82 mango volatiles in *Bactrocera dorsalis*: From structural analysis to behavioral validation. Front. Insect Sci..

[47] Mustapha T., Zhang Y., Yan J., Tang H., Wang Z., Wu S.Y., Basit A., Boulaamane Y., Chandra A., Abdullahi M.A.I. (2026). Odorant-Binding Protein Interactions with Herbivore-Induced Volatiles Drive Behavioral Attraction of *Harmonia axyridis* (Coleoptera: Coccinellidae) to Tuta Absoluta-Infested Tomato Plant. J. Agric. Food Chem..

[48] Gilson M.K., Zhou H.X. (2007). Calculation of protein-ligand binding affinities. Annu. Rev. Biophys. Biomol. Struct..

[49] Pelosi P., Zhou J.J., Ban L., Calvello M. (2006). Soluble proteins in insect chemical communication. Cell. Mol. Life Sci..

[50] Du X., Li Y., Xia Y.L., Ai S.M., Liang J., Sang P., Ji X.L., Liu S.Q. (2016). Insights into protein–ligand interactions: Mechanisms, models, and methods. Int. J. Mol. Sci..

[51] Bourdon P.A., Zottele M., Zafar Z., Baxter I., Midthassel A., Myrta A., Wechselberger K.F., Strasser H., Butt T.M. (2023). Behavioral response of three subterranean pests (*Agriotes lineatus*, *Diabrotica virgifera virgifera*, *Phyllopertha horticola*) to the fungal volatile organic compounds 1-octen-3-ol and 3-octanone. Arthropod Plant Interact..

