# Supplementary material for: Molecular Recognition Mechanism of Key VOCs by Odorant-Binding Proteins in the Western Corn Rootworm (Diabrotica virgifera virgifera)
Source: Insects. 2026 Jun 5;17(6):595. doi: 10.3390/insects17060595 (PMC13300332; doi:10.3390/insects17060595)
Supplement: Supplementary file 1 [file insects-17-00595-s001.zip › insects-4295745-supplementary.pdf]

Figure S1. Homology models of representative *D. v. virgifera* odorant-binding proteins.

Figure S2. Ramachandran plot validation for the structural models of four representative DvirOBP subclasses.

Figure S3. Local intramolecular stabilization networks within the DvirOBP54b pocket.

Figure S4. Results of the 50 ns Molecular Dynamics Simulation.

Figure S5. Scatchard plot analysis of 1-NPN binding to three representative DvirOBPs.

Table S1. Intramolecular interactions of DvirOBP54b with 6-MBOA, (E)-β-caryophyllene, and 2R,8R-MDP.

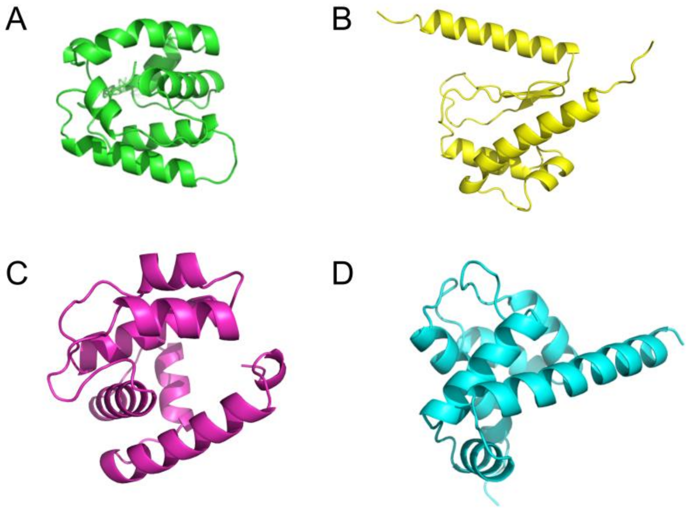

Figure S1. Homology models of representative *D. v. virgifera* odorant-binding proteins. (A) DvirOBP53, (B) DvirOBP54a, (C) DvirOBP54b, and (D) DvirOBP55.

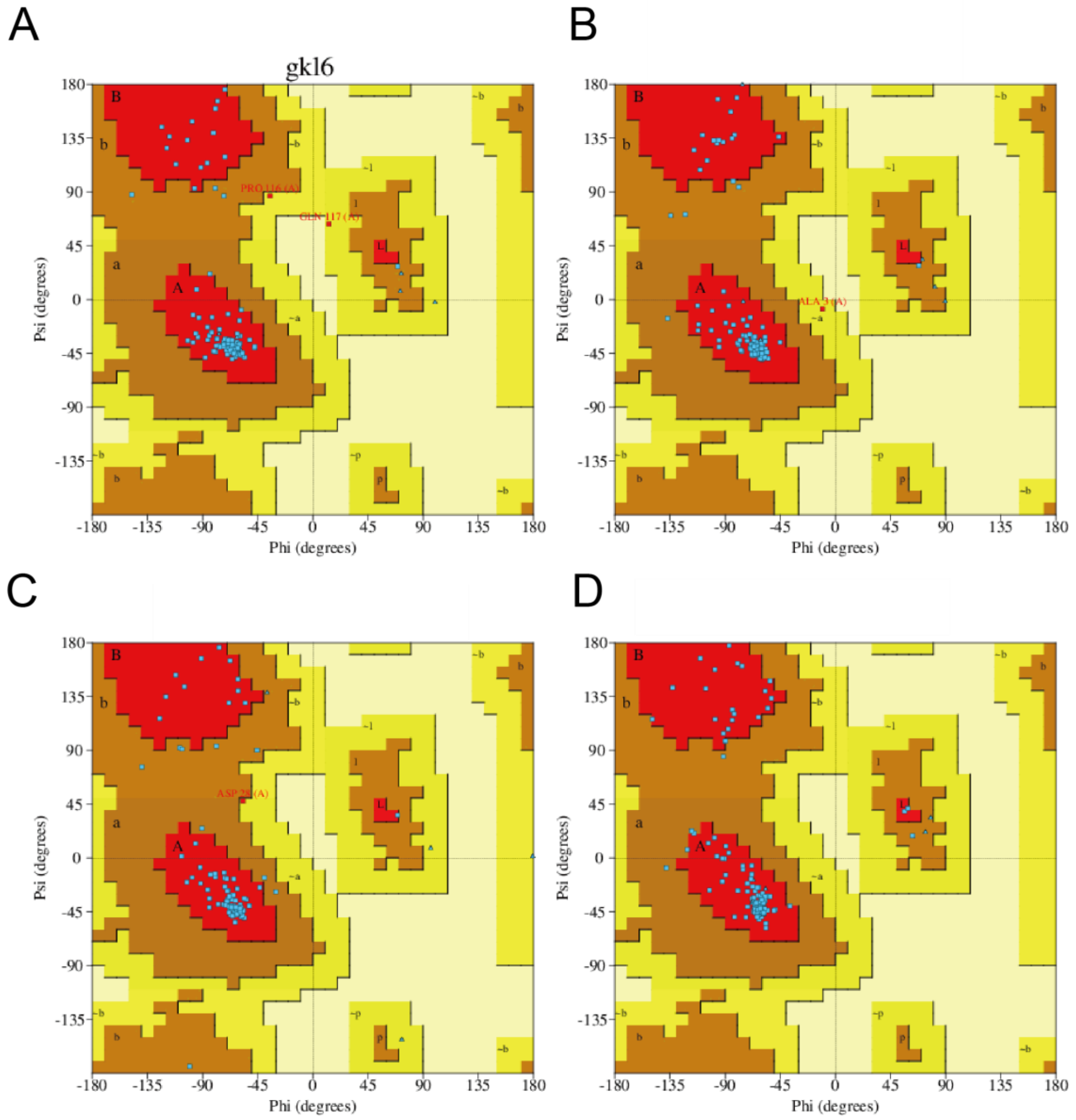

Figure S2. Ramachandran plot validation for the structural models of four representative DvirOBP subclasses. Stereochemical quality of (A) DvirOBP53, (B) DvirOBP54a, (C) DvirOBP54b, and (D) DvirOBP55 was validated using PROCHECK. The color-coded regions define the conformational spaces for backbone dihedral angles: red indicates core favored regions; brown indicates additionally allowed regions; yellow indicates generously allowed regions; and pale-yellow represents disallowed regions. The dominant clustering of amino acid residues (blue dots) within the favored and allowed zones confirms the high stereochemical fidelity of these representative odorant-binding protein models.

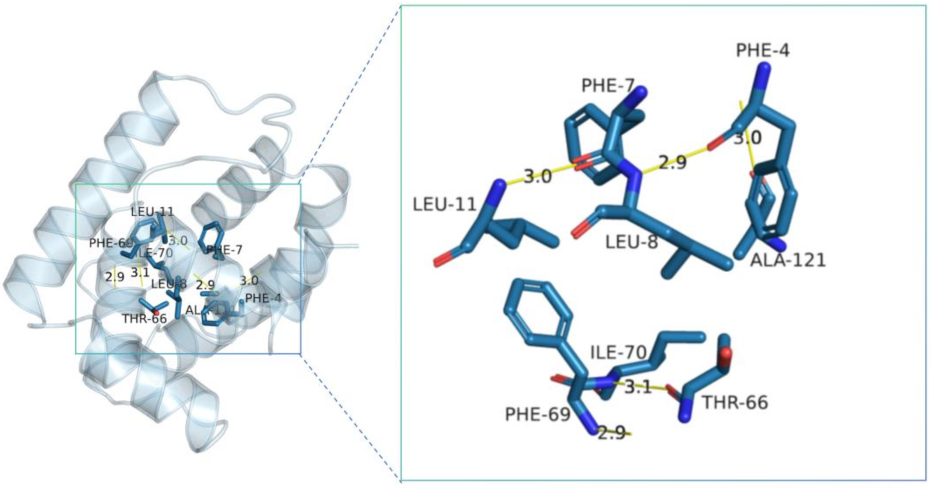

.

Figure S3. Local intramolecular stabilization networks within the DvirOBP54b pocket. Dashed lines represent hydrogen bonds with precise atomic distances (Å) monitored between proximal residues flanking the dynamic binding tunnel.

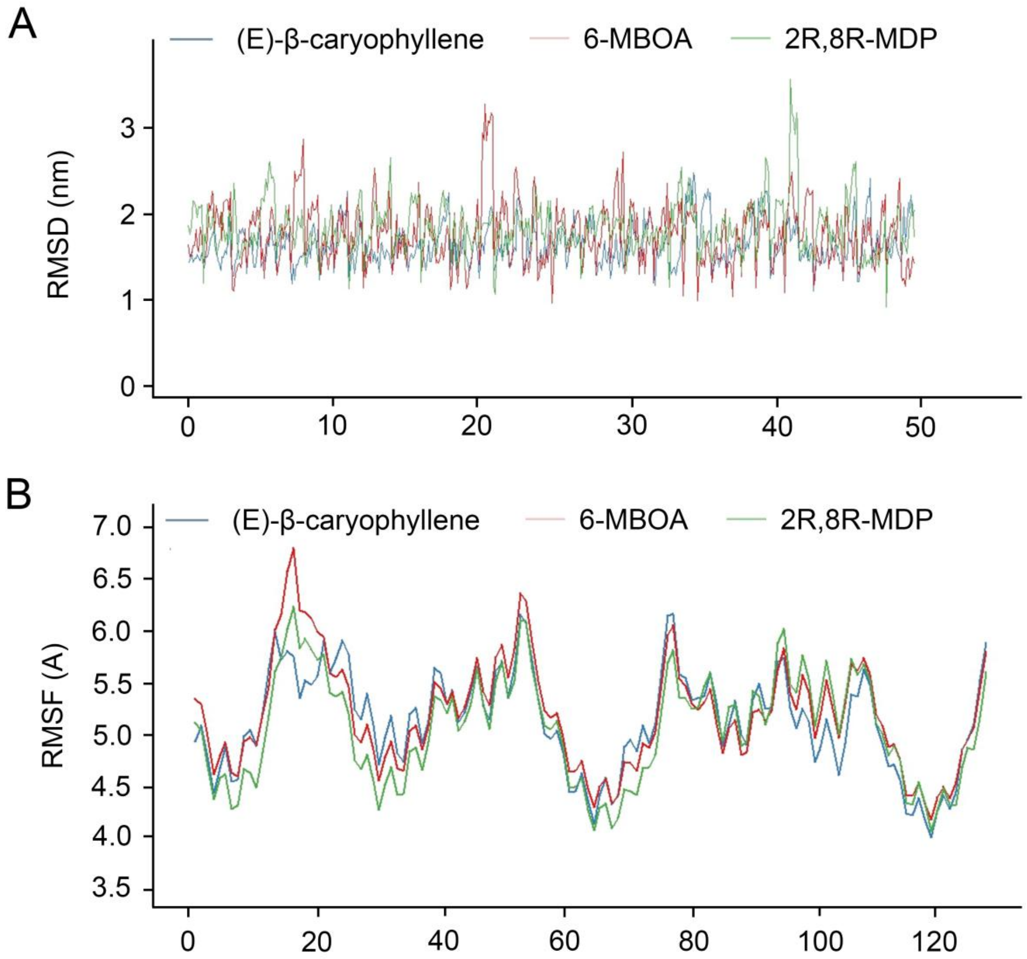

Figure S4. Results of the 50 ns Molecular Dynamics Simulation. (A) RMSD time trajectories (0–50 ns) of the three complexes; (B) RMSF profiles of residue fluctuations.

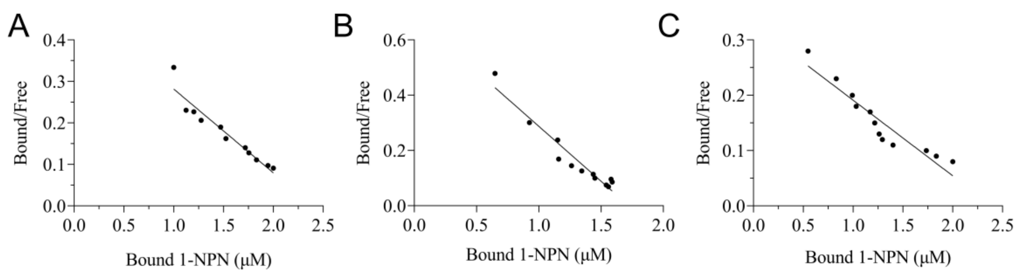

Figure S5. Scatchard plot analysis of 1-NPN binding to three representative DvirOBPs. The plots for (A) DvirOBP53, (B) DvirOBP54a, and (C) DvirOBP54b were derived from the corresponding fluorescence competitive binding data.

Table S1. Intramolecular interactions of DvirOBP54b with 6-MBOA, (E)-β-caryophyllene, and 2R,8R-MDP.

DvirOBP54b-6-MBOA			DvirOBP54b-(E)-β-caryophyllene			DvirOBP54b-2R,8R-MDP		
Interaction type	Residue	Distance	Interaction type	Residue	Distance	Interaction type	Residue	Distance
Hydrophobic Interactions	PHE4	3.65	Hydrophobic Interactions	PHE4	3.96	Hydrophobic Interactions	PHE7	3.91
Hydrophobic Interactions	PHE7	3.30	Hydrophobic Interactions	PHE7	3.25	Hydrophobic Interactions	PHE69	3.73
Hydrophobic Interactions	LEU8	3.66	Hydrophobic Interactions	PHE69	3.26	Hydrophobic Interactions	ILE70	3.62
Hydrophobic Interactions	LEU11	3.72	Hydrophobic Interactions	ILE70	3.50	Hydrophobic Interactions	THR73	3.94
Hydrophobic Interactions	THR66	3.68	Hydrophobic Interactions	THR73	3.79	Hydrophobic Interactions	LEU75	3.87
Hydrophobic Interactions	PHE69	3.65	Hydrophobic Interactions	LEU75	3.86	Hydrophobic Interactions	PHE91	3.80
Hydrophobic Interactions	ILE70	3.33	Hydrophobic Interactions	PHE91	3.87	Hydrophobic Interactions	ALA121	3.67
Hydrophobic Interactions	ALA121	3.74	Hydrophobic Interactions	ALA121	3.23	Hydrophobic Interactions	VAL124	3.99
Hydrogen Bonds	THR66	3.42	Hydrophobic Interactions	VAL125	3.84			

Note: Interaction types, key binding residues, and atomic distances (Å) modeled within the dynamic olfactory pocket of representative representative DvirOBP54b.
